# Supplementary material for: Laboratory Risk Assessment of Three Entomopathogenic Fungi Used for Pest Control toward Social Bee Pollinators
Source: Microorganisms. 2022 Sep 7;10(9):1800. doi: 10.3390/microorganisms10091800 (PMC9501116; doi:10.3390/microorganisms10091800)
Supplement: Supplementary file 1 [file microorganisms-10-01800-s001.zip › microorganisms-1849230-supplementary.pdf]

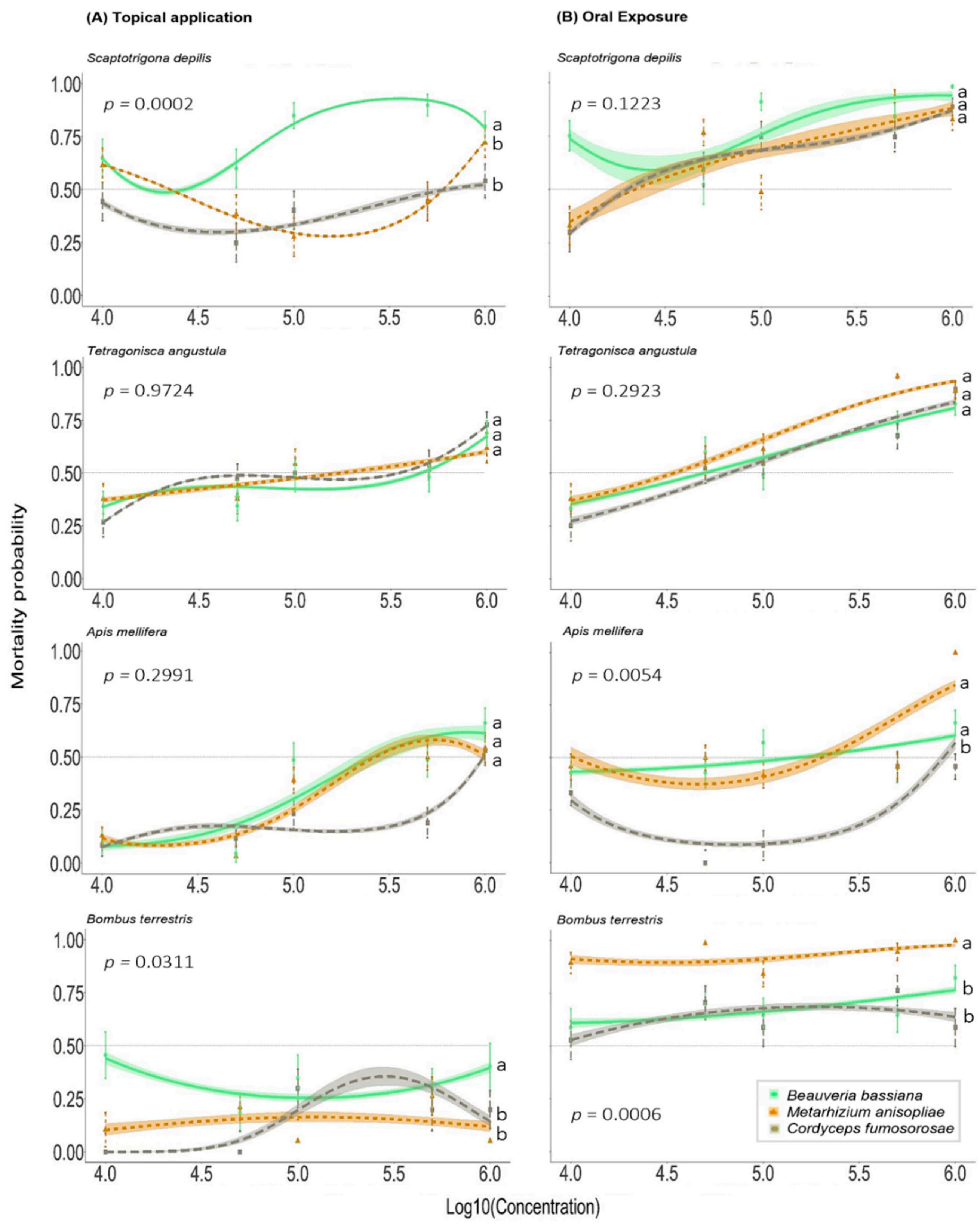

Figure S1. Corrected mortality probability of *Scaptotrigona depilis*, *Tetragonisca angustula*, *Apis mellifera* and *Bombus terrestris* workers by the entomopathogenic fungi *Beauveria bassiana*, *Metarhizium anisopliae* and *Cordyceps fumosorosea* by (**A**) topical and (**B**) oral exposures. The concentrations (conidia mL⁻¹) were transformed by regression Log10: 4.0 = 5 × 105, 4.5 = 1 × 106, 5.0 = 5 × 106, 5.5 = 1 × 107, 6.0 = 5 × 107. Different letters compare the mean mortality of all concentrations and indicate that there was significant difference between the results ($p < 0.05$).
